# Polychlorinated Alkanes in Fish from Norwegian Freshwater

**DOI:** 10.1100/tsw.2002.82

**Published:** 2002-01-16

**Authors:** Anders R. Borgen, Martin Schlabach, Roland Kallenborn, Eirik Fjeld

**Affiliations:** Norwegian Institute for Air Research, Instituttveien 18, P.O. Box 100, N-2027 Kjeller, Norway; Norwegian Institute for Air Research, Polarmiljøsenteret, Hjalmar Johansens gt. 14, N-9296 Tromsø, Norway; Norwegian Institute for Water Research, Brekkeveien 19, P.O. Box 173, Kjelsås, 0411 Oslo, Norway

**Keywords:** chlorinated paraffins, ECNI, freshwater fish, HRGC/HRMS

## Abstract

Short-chain polychlorinated alkanes (sPCAs) have been measured in freshwater fish samples from different lakes all over Norway and from the Norwegian Arctic. The analyses were performed with high-resolution GC coupled to high-resolution MS in electron capture negative ion mode. The species investigated were trout, Arctic char, and burbot (Lota lota). Muscle tissue in the lake trout and Arctic char, and liver in burbot, were selected for analyses because of their high lipid content. ∑sPCA concentration ranged from 108 to 3700 ng/g fat. The highest value was found in the south of Norway near an industrial area.

## INTRODUCTION

Polychlorinated alkanes (PCA), or chlorinated paraffins, are straight-chain alkanes with varying degrees of chlorination. They have been produced since the 1930s to an extent of approximately 300 kilotons estimated for the western world[1]. PCA are mainly produced by direct chlorination of a petroleum fraction with molecular chlorine in the presence of UV light[2].
PCA have been used as additives in high temperature and pressure lubricants as well as secondary plasticisers and flame retardants in plastics and paints[1,4].

PCA are divided into three main categories — short- (C10-C13), medium- (C14-C17), and long-chain (C18-C30) — and further by their degree of chlorination — low (<50%) and high (>50%)[4]. Because of their relatively high assimilation and accumulation potential, the short-chain, highly chlorinated PCAs have been most widely studied. Although PCA generally have shown low toxicity to mammals, short-chain PCA (sPCA) have a carcinogenic potential in rats and mice[5]. In addition, recent dose-response studies have shown that oral intake of sPCA by mice results in an increase in liver weight, which is considerable compared to reference materials[12]. They have also been shown to be toxic towards certain species in the aquatic environment[4,6,7], although at concentration levels several orders of magnitude higher than for TCDD[7].

The complexity of sPCA mixtures make it difficult to provide an analytical method for their precise and specific quantitative determination. Technical sPCA mixtures consist of several thousand components, and due to the large number of isomers, complete chromatographic separation seems impossible at this point. This analytical challenge has resulted in different analytical approaches to analysis of sPCA[1,2,3,5,8,9,10,17,18].

In this study, sPCA have been measured in lake trout, burbot, and Arctic char from different locations in Norway, and in the Norwegian Arctic.

## EXPERIMENTAL

The fish samples were homogenised with sodium sulphate and 13C-labelled PCB 118 was added as an internal standard. The samples were then extracted with a mixture of ethyl acetate and cyclohexane (1:1). Separation of the PCAs from the fat was done on a GPC system with SX-3 Bio-Beads, eluted with a mixture of ethyl acetate and cyclohexane (1:1) at 5 ml/min. The fraction containing sPCA was further cleaned on a column packed with 30 g of aluminum oxide. Reduction of the sample volumes to about 100 µl were done on a Zymark TurboVap 500, and the samples were at this point ready for analysis on HRGC/HRMS.

An HP5890 GC coupled to a VG AutoSpec high-resolution MS was used for all of the analyses. The MS was operated in electron capture negative ion mode with methane at a pressure of $2 \times 10^{-5}$ mbar as reagent gas. The GC was operated in constant flow mode, 1 ml/min, with a temperature program starting at 150°C, then ramping to 260°C by 7°C/min. The temperature was held at 260°C for 8 min and was then increased to 280°C by 10°C/min, holding that temperature for 13 min. The injector temperature was 260°C. Quantification was performed according to the method described by Tomy et al[3].

Due to limitations in the AutoSpec software, previous sPCA analyses had to be done using three injections for each sample[14]. This problem has now been rectified and as a result the GC/MS program used in this study has been adapted to one injection per analysis.

## RESULTS AND DISCUSSION

In Table 1, the results are reported as the sum (Σ) of C10–C13 PCAs with five to ten chlorine substitutions. Calculated average molar masses are indicative of the PCA formula group profiles observed in the samples. The lipid content of each sample is also reported.

The burbot liver samples seem to contain sPCA with a higher average molar mass than the trout muscle samples. This may be due to a higher rate of biotransformation in the liver, and that sPCA with lower chlorine content seem to be more easily metabolised than the higher chlorinated sPCA[5]. Another possible explanation is that the burbot are generally at a higher trophical level than the trout, which may result in intake of PCA with higher average molar mass. The size of the trout may also be important for its predatory abilities, and therefore also the individual placement in the food chain. This may again be one of the explanations of the variations in the average molar mass of the trout samples.

The sPCA pattern (Fig. 1) and the average molar mass of the sample from Lake Ellasjøen, located on Bjørnøya Island (74° N), is quite high considering the location of this lake (compare with Fig. 2, sPCA pattern from a technical mixture with 55.5% Cl). With only long-range transport by air, the average molar mass would be expected to be lower than in samples collected near PCA sources. Lake Ellasjøen is located in the catchment area of large nesting colonies of seabirds, and the result of the analysis of this Arctic char sample supports the theory of persistent organic pollutants being transported to Lake Ellasjøen by seabirds together with long range transport by air[13].

**TABLE 1**
**Concentrations and Calculated Average Molar Masses of Short-Chained and Highly Chlorinated PCA in Freshwater Fish Samples from Different Locations in Norway**

| Location | Sample Type | ΣsPCA in ng/g fat | Lipid Content (%) | Average Molar Mass, g/mole |
|---|---|---|---|---|
| Takvatn | Trout, muscle | 172 | 1.80 | 396 |
| Fjellfrøsvatnet | Trout, muscle | 545 | 1.10 | 378 |
| Grunnvatnet | Trout, muscle | 1692 | 1.30 | 421 |
| Store Raudvannet | Trout, muscle | 108 | 2.50 | 411 |
| Selbusjøen | Trout, muscle | 436 | 1.40 | 389 |
| Breimsvatn | Trout, muscle | 923 | 1.30 | 427 |
| Bogevatnet | Trout, muscle | 1414 | 0.70 | 395 |
| Kalsjøen | Trout, muscle | 178 | 1.80 | 394 |
| Kalandsvatn | Trout, muscle | 254 | 2.60 | 387 |
| Vegår | Trout, muscle | 263 | 1.90 | 407 |
| Mårvann | Trout, muscle | 256 | 1.60 | 415 |
| Grindheimsvatn | Trout, muscle | 733 | 0.90 | 394 |
| Lygne | Trout, muscle | 408 | 1.30 | 408 |
| Ellasjøen | Arctic char, muscle | 592 | 1.30 | 453 |
| Velmunden | Arctic char, muscle | 500 | 1.00 | 435 |
| Grensefoss | Burbot, liver | 741 | 11.6 | 435 |
| Selbusjøen | Burbot, liver | 226 | 38.5 | 421 |
| Røgden | Burbot, liver | 787 | 34.8 | 456 |
| Røgden | Burbot, liver | 1152 | 34.3 | 422 |
| Øgderen | Burbot, liver | 695 | 22.0 | 417 |
| Femsjøen | Burbot, liver | 3700 | 40.0 | 429 |
| PCA 55.5 % | Standard | | | 407 |

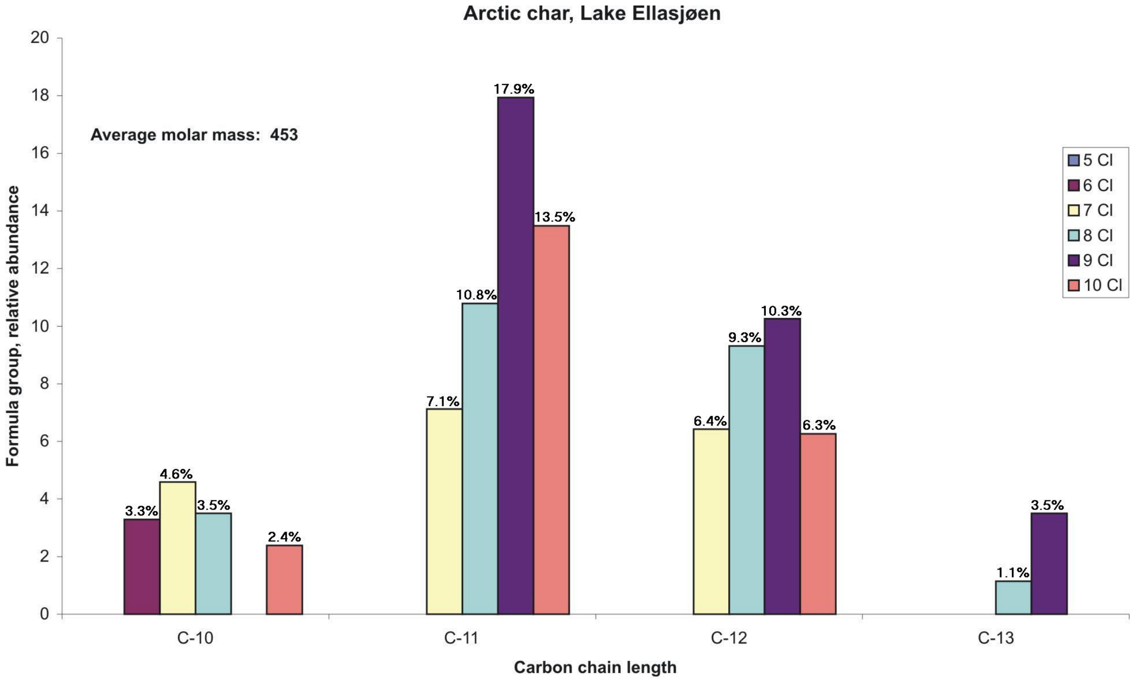

**FIGURE 1.** sPCA pattern in the Arctic char sample from Lake Ellasjøen.

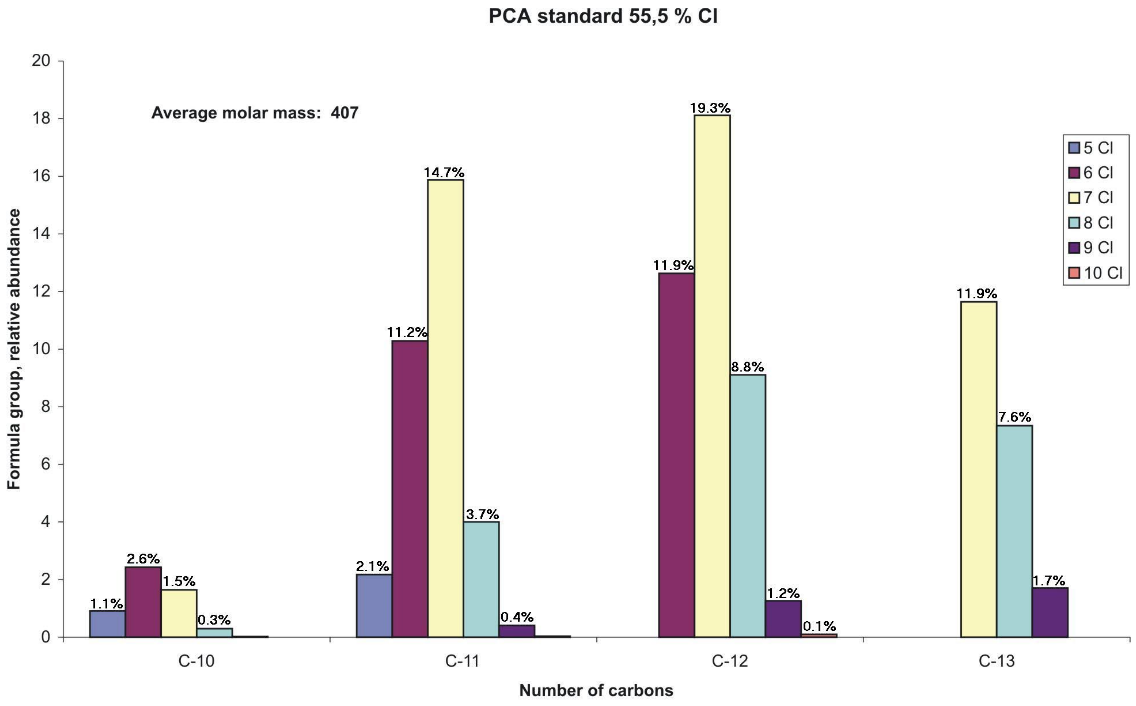

**FIGURE 2.** sPCA pattern in a technical mixture containing 55.5% Cl used as a quantification standard.

The concentration of sPCA in the sample from Lake Femsjøen, located near the Swedish border in the south of Norway, is of the same order of magnitude as in an Arctic char sample collected in the south of Sweden[15].

The concentrations of sPCA in lake trout, reported here, are about an order of magnitude lower than concentrations of rainbow trout samples collected in Lake Ontario, Canada[16].

## ACKNOWLEDGEMENT

Anders R. Borgen would like to thank The Research Council of Norway for financing this project, and Jeremy R. Hart for providing VMS software information.
